# Crystal Structure of the Cysteine Desulfurase DndA from *Streptomyces lividans* Which Is Involved in DNA Phosphorothioation

**DOI:** 10.1371/journal.pone.0036635

**Published:** 2012-05-03

**Authors:** Fukun Chen, Zhenyi Zhang, Kui Lin, Tianle Qian, Yan Zhang, Delin You, Xinyi He, Zhijun Wang, Jingdan Liang, Zixin Deng, Geng Wu

**Affiliations:** State Key Laboratory of Microbial Metabolism, School of Life Sciences and Biotechnology, Shanghai Jiao Tong University, Shanghai, China; National Institute for Medical Research, Medical Research Council, United Kingdom

## Abstract

DNA phosphorothioation is widespread among prokaryotes, and might function to restrict gene transfer among different kinds of bacteria. There has been little investigation into the structural mechanism of the DNA phosphorothioation process. DndA is a cysteine desulfurase which is involved in the first step of DNA phosphorothioation. In this study, we determined the crystal structure of *Streptomyces lividans* DndA in complex with its covalently bound cofactor PLP, to a resolution of 2.4 Å. Our structure reveals the molecular mechanism that DndA employs to recognize its cofactor PLP, and suggests the potential binding site for the substrate L-cysteine on DndA. In contrast to previously determined structures of cysteine desulfurases, the catalytic cysteine of DndA was found to reside on a β strand. This catalytic cysteine is very far away from the presumable location of the substrate, suggesting that a conformational change of DndA is required during the catalysis process to bring the catalytic cysteine close to the substrate cysteine. Moreover, our in vitro enzymatic assay results suggested that this conformational change is unlikely to be a simple result of random thermal motion, since moving the catalytic cysteine two residues forward or backward in the primary sequence completely disabled the cysteine desulfurase activity of DndA.

## Introduction

DNA phosphorothioation is a unique type of epigenetic modification which occurs on the DNA backbone. During this process, a sulfur atom replaces a non-bridging oxygen atom of the phosphodiester backbone of DNA. DNA phosphorothioation was first discovered in *Streptomyces lividans* [1], [2], and was later found to widely exist in many other kinds of bacteria [3], [4]. The biological consequences of DNA phosphorothioation have not been fully understood, though it has been suggested that it might function to restrict transfer of genetic materials among different species of bacteria [5].

Five proteins (DndA, DndB, DndC, DndD, and DndE) encoded by the *dnd* gene locus are necessary and sufficient for the process of DNA phosphorothioation in *Streptomyces lividans* [2], [6]. Among these five proteins, DndA has been found to be a cysteine desulfurase, catalyzing the removal of sulfur from the substrate L-cysteine and reconstituting the iron-sulfur cluster in DndC. Therefore, it has been suggested that the DndA-catalyzed sulfur mobilization is the first step during the DNA phosphorothioation procedure [7].

Cysteine desulfurases are involved in the syntheses of many kinds of sulfur-containing biomolecules, such as assemblies of iron-sulfur clusters in proteins involved in nitrogen fixation, syntheses of thiamin and molybdopterin cofactors, and formation of thionucleosides in tRNAs [8], [9]. IscS (for iron-sulfur cluster), NifS (for nitrogen fixation), and SufS (for sulfur utilization) are typical cysteine desulfurases which have been studied most extensively [10]–[12]. They all catalyze the formation of L-alanine and elemental sulfur from the substrate L-cysteine. CsdB, a selenocysteine lyase, is related to cysteine desulfurases. However, it is specific for L-selenocysteine and catalyzes its decomposition into L-alanine and selenium [13]. IscS, NifS, SufS, and CsdB all contain a tightly bound pyridoxal 5′-phosphate (PLP) cofactor, which forms Schiff base with a highly conserved lysine residue. In addition, they all have an active site cysteine which is essential for their desulfurization/deselenoation reaction. This catalytic cysteine uses its thiolate anion to attack the sulfur atom of the substrate cysteine, and forms a persulfide bond with it. Crystal structures of *Escherichia coli* IscS [14, PDB code 1P3W], *Thermotoga maritima* NifS [15, PDB code 1ECX], *Synechocystis* sp. PCC 6803 SufS [16, PDB code 1T3I], and *Escherichia coli* CsdB [17–18, PDB code 1C0N] have been solved. In these structures, the catalytic cysteines were found to exist either on a long and flexible loop (in the cases of IscS and NifS), or on a short loop (in the cases of SufS and CsdB).

Up to now, there has been not much structural investigation on proteins involved in DNA phosphorothioation [19]. In this study, we determined the crystal structure of DndA from *Streptomyces lividans*, to a resolution of 2.4 Å. Our structure reveals the molecular mechanism that DndA employs to recognize the PLP cofactor. Moreover, the catalytic cysteine of DndA, Cys327, was found to exist on a β strand, different from other cysteine desulfurases. In addition, our in vitro biochemical cysteine desulfurase activity assay demonstrates that the position of this catalytic cysteine is essential for its activity, and moving it two residues forward or backward on the primary sequence completely disabled its cysteine desulfurase activity.

## Results

### Structure determination and overall structure of DndA

To understand the molecular basis of how DndA performs its function, we tried crystallization on both full length wild type (WT) *Streptomyces lividans* DndA and a C327S point mutant in which the catalytic cysteine, Cys327, was replaced by a serine. Despite considerable effort, we were not able to obtain crystals for WT DndA, presumably due to protein heterogeneity caused by oxidation of the highly reductive Cys327. On the other hand, we successfully crystallized the C327S mutant of DndA (referred to as DndA hereafter), and determined its crystal structure to a resolution of 2.4 Å (Table 1).

**Table 1:** Data collection and refinement statistics.

Parameter	Values
**Data collection**	
Space group	*P*2
Wavelength (Å)	0.97915
Unit cell parameters	a = 77.9 Å, b = 67.3 Å, c = 85.6 Å, β = 97.0°
Number of molecules/asymmetric unit	2
Resolution (Å) (outer shell)	45-2.40 (2.53-2.40)
Oscillation range per frame (°)	1
Crystal mosaicity	0.51
Data multiplicity (outer shell)	7.3 (7.4)
Matthews coefficient (Å^3^Da^−1^)	2.78
Solvent content (%)	55.81
Total reflections	251,126
Unique reflections	34,563
I/σ_I_ (outer shell)	3.9 (1.7)
Completeness (%) (outer shell)	99.8 (100.0)
R_merge_ (%) (outer shell)	14.7 (41.0)
**Refinement**	
Number of reflections used	32,825
R_work_ (%)	19.2
R_free_ (%)	23.4
B-factor (Å^2^)	
Overall	27.9
protein	27.8
ligand	17.8
solvent	31.5
RMSD bond lengths (Å)	0.008
RMSD bond angles (°)	1.117
RMSD B factor of main chain bond (Å^2^)	0.907
RMSD B factor of side chain bond (Å^2^)	1.486
Final model (number of protein atoms)	5,472
Final model (number of water atoms)	269

R_merge_ = Σ_h_Σ_i_ |*I*_h,i_−*I*_h_|/Σ_h_Σ_i_ *I*_h,i_ for the intensity (*I*) of i observation of reflection h. R factor = Σ∥*F*_obs_|−|*F*_calc_∥/Σ|*F*_obs_|, where *F*_obs_ and *F*_calc_ are the observed and calculated structure factors, respectively. R_free_ = R factor calculated using 5% of the reflection data chosen randomly and omitted from the start of refinement. RMSD, root-mean-square deviations from ideal geometry. Data for the highest resolution shell are shown in parentheses.

**Figure 1:**
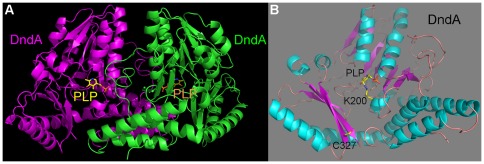
Crystal structure of DndA from *Streptomyces lividans*. (**A**) **Overall structure of the DndA dimer.** The structure is viewed perpendicular to the two-fold axis of the dimer. The two protomers are shown in magenta and green, respectively. Their bound PLP cofactors are presented as sticks, with carbon atoms yellow, nitrogen atoms blue, oxygen atoms red, and phosphorus atoms orange. (**B**) **Structure of a protomer of DndA.** α helices are shown in cyan, β sheets are shown in magenta, and loops are shown in pink. PLP and its covalently linked Lys200 of DndA, as well as the catalytic Cys327 (mutated to serine in our study), are shown in stick representation.

In the crystal structure, DndA forms a homodimer in which the two protomers are related by a two-fold rotation axis (Figure 1A), similar to other cysteine desulfurases IscS, NifS, SufS, and CsdB [14]–[18]. In each protomer, there is a PLP cofactor which forms a Schiff base covalent bond with the amino group of Lys200 (Figure 1B).

**Figure 2:**
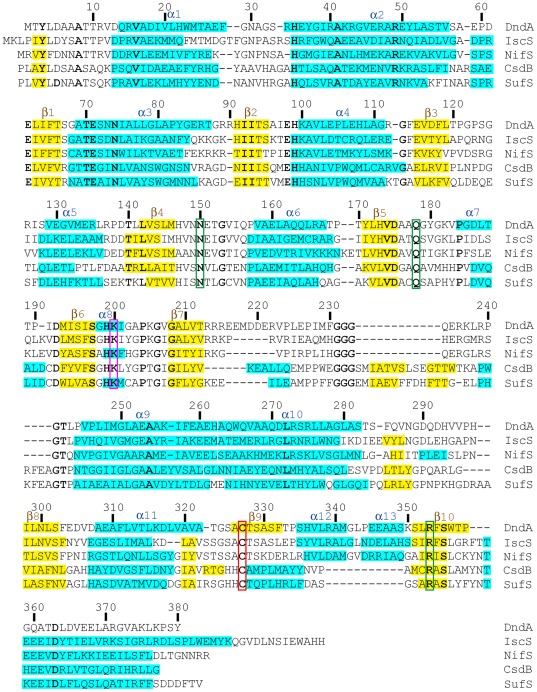
Structure-based sequence alignment of DndA and related cysteine desulfurases/selenocysteine lyase. The amino acid sequences of DndA from *Streptomyces lividans*, IscS from *Escherichia coli*, NifS from *Thermotoga maritima*, CsdB from *Escherichia coli*, and SufS from *Synechocystis* sp. PCC 6803 are aligned based on their sequence homology and secondary structures. α helices are shaded in cyan, and β sheets are shaded in yellow. Residues conserved in all five proteins are shown in bold letters. Secondary structure elements and residue numbers for DndA are shown above the sequences. The conserved catalytic cysteine residues (Cys327 in DndA) are emphasized with a red box. The conserved lysine residues (Lys200 in DndA) which form Schiff base covalent links with bound PLP's are indicated with a magenta box. The residues which are presumed to recognize the carboxylate group of L-cysteine substrates (Asn150, Gln178, and Arg353 in DndA) are marked with green boxes.

Each protomer of DndA consists of a larger N-terminal domain and a smaller C-terminal domain (Figure 1B). The larger domain (residues 1–262), where the PLP cofactor resides on, is more conserved among cysteine desulfurases (Figure 2). It mainly consists of a seven-stranded β sheet, flanked by α helices on both sides. The smaller domain (residues 263–380) exhibits greater variance and has only a few conserved residues, including Cys327. It is composed of a three-stranded β sheet and four α helices (Figure 1B).

### The interaction interface between DndA and PLP

**Figure 3:**
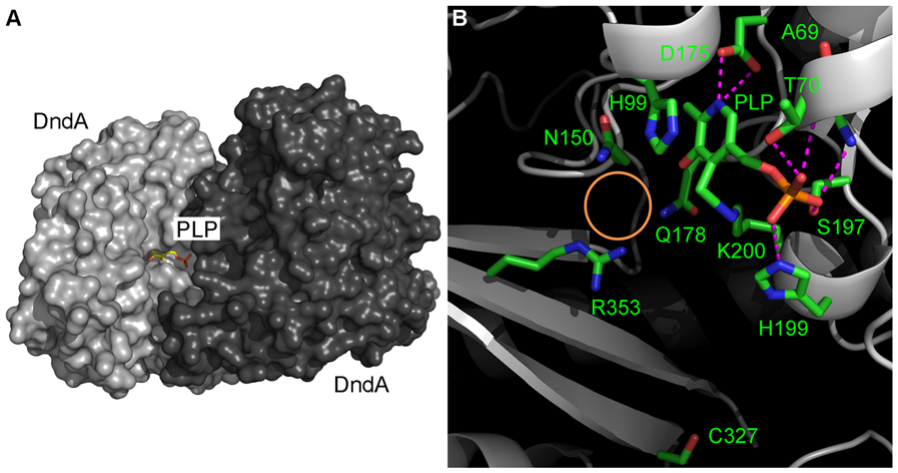
The binding site of PLP on DndA. (**A**) **PLP is located in a deep surface pocket on DndA.** The two protomers of DndA are shown in surface representation, with only one PLP shown in stick representation. The protomer of DndA harboring this PLP is colored in light grey, whereas the other protomer is colored in dark grey. Blue, red, yellow, and orange represent nitrogen, oxygen, carbon, and phosphorus atoms, respectively. (**B**) **The interaction interface between PLP and DndA.** DndA is shown in grey, with carbon atoms of its side chains and PLP shown in green. Blue, red, and orange represent nitrogen, oxygen, and phosphorus atoms, respectively. Hydrogen bonds are represented by magenta dashed lines. The orange circle indicates the presumable location of the carboxylate group of the L-cysteine substrate.

Similar to other cysteine desulfurases, DndA uses a deep surface pocket to harbor the PLP cofactor (Figure 3A). Besides Lys200 which forms a Schiff base with PLP, there are many other residues forming hydrogen bonds and van der Waals interactions with it (Figure 3B). For example, His99 uses its imidazole ring to stack on top of the pyridine ring of PLP, making multiple van der Waals interactions with it. In addition, Asp175, which is at the bottom of the surface pocket, forms a couple of hydrogen bonds with the pyridine N1 atom of PLP. Moreover, the main chain of Ala69, the main chain and side chain of Thr70, and the side chains of Ser197 and His199 hydrogen bond to the phosphate group of PLP (Figure 3B). Interestingly, Thr70, His99, Asp175, Ser197, His199, and Lys200 are all strictly conserved among all the cysteine desulfurases/selenocysteine lyases we examined (Figure 2 and data not shown) [3]. Therefore, DndA uses multiple interactions to fix PLP at this position, so that it would not dissociate from its binding site even when its Schiff base with Lys200 is broken in exchange of forming a Schiff base with the amino group of the substrate L-cysteine.

### Potential binding site for the substrate L-cysteine

Despite considerable effort, we were not able to obtain co-crystals of DndA in complex with the substrate L-cysteine. However, by comparison with structures of other cysteine desulfurases/selenocysteine lyase, we can infer where the binding site for the substrate L-cysteine is on DndA.

When the substrate L-cysteine enters, its amino group will make a Schiff base with the aldehyde group of PLP, in exchange of the Schiff base between the amino group of Lys200 and PLP [15]. In addition, it has been suggested that Arg379 and Asn175 in CsdB probably serve as recognition residues for the carboxylate group of the substrate L-selenocysteine [17], [18]. Both Arg379 and Asn175 in CsdB are conserved in DndA, and they correspond to DndA residues Arg353 and Asn150 which are in close vicinity to PLP. The guanidine group of Arg353, the amino group of Asn150, and the amino group of the nearby Gln178 all carry positive charges, and all point to the same direction (the orange circle in Figure 3B). Importantly, Asn150, Gln178, and Arg353 are stringently conserved among all the cysteine desulfurases/selenocysteine lyases we examined (Figure 2 and data not shown). Therefore, we hypothesize that the empty space surrounded by Asn150, Gln178, and Arg353 (the orange circle in Figure 3B) is the binding site for the carboxylate group of the substrate L-cysteine.

### The catalytic cysteine of DndA, Cys327, resides on a β strand

In contrast to previously determined structures of cysteine desulfurases/selenocysteine lyase, in which the catalytic cysteines are all located on flexible loops, the catalytic cysteine Cys327 of DndA (mutated to serine in our study) was found to reside on a β strand (Figure 2 and Figure 3B). It is on the surface of the DndA protein, and its side chain is pointing into the solvent. Therefore, we do not believe that this structural difference was caused by the cysteine to serine mutation. The distance between Cys327 and PLP is about 16 Å (Figure 1B), and the distance between Cys327 and the presumable position of L-cysteine substrate is also more than 10 Å (Figure 3B). Therefore, a dramatic conformational change is needed for Cys327 to approach near the L-cysteine substrate to carry out the nucleophilic attack.

### The position of the catalytic cysteine is essential for the cysteine desulfurase activity of DndA

It has been suggested that the flexibility of the loop containing the catalytic cysteine might be the reason why the catalytic cysteine can overcome the long distance to attack the substrate L-cysteine [14]. If this flexibility simply results from a random thermal motion, then residues close to the catalytic cysteine in the primary sequence should also have certain chances to approach the substrate cysteine. Under this circumstance, moving the catalytic cysteine to a nearby position on the primary sequence would not expect to cause a disastrous effect, and should retain some of the cysteine desulfurase activity. To test whether this possibility is true for DndA, we created two double point mutants of DndA, S325C/C327S and C327S/S329C, with the apparent result that the position of the catalytic cysteine was moved two residues forward or backward on the primary sequence. We chose Ser325 and Ser329 to mutate for two reasons. First, the side chain of serine is most similar to cysteine; and second, the residues one spacing (Thr328) or three spacings (Ala330) forward are on the opposite side of the β strand.

**Figure 4:**
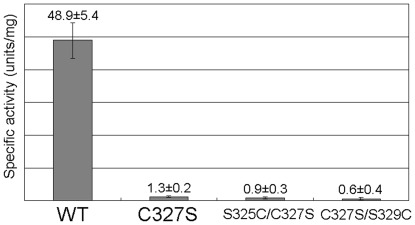
Specific activities of WT and point mutants of DndA measured using in vitro cysteine desulfurase activity assay. Assays were performed for five times, and the average values of specific activities along with standard deviations of the measurements were shown.

When we performed the in vitro enzymatic activity assay, WT DndA exhibits high specific activity (48.9 units/mg), similar to that previously reported (38.6 units/mg) [7] and comparable with those reported for *E. coli* IscS (78 units/mg) [10] and *A. vinelandii* NifS (89 units/mg) [20]. In contrast, the C327S mutant of DndA completely lost the cysteine desulfurase activity (1.3 units/mg). Importantly, the S325C/C327S (0.9 units/mg) and C327S/S329C (0.6 units/mg) double point mutations also drastically eliminated the cysteine desulfurase activity of DndA (Figure 4). Therefore, we conclude that the conformational change of DndA which brings the catalytic cysteine to the substrate cysteine is unlikely to be a simple result from random thermal motion.

## Discussion

**Figure 5:**
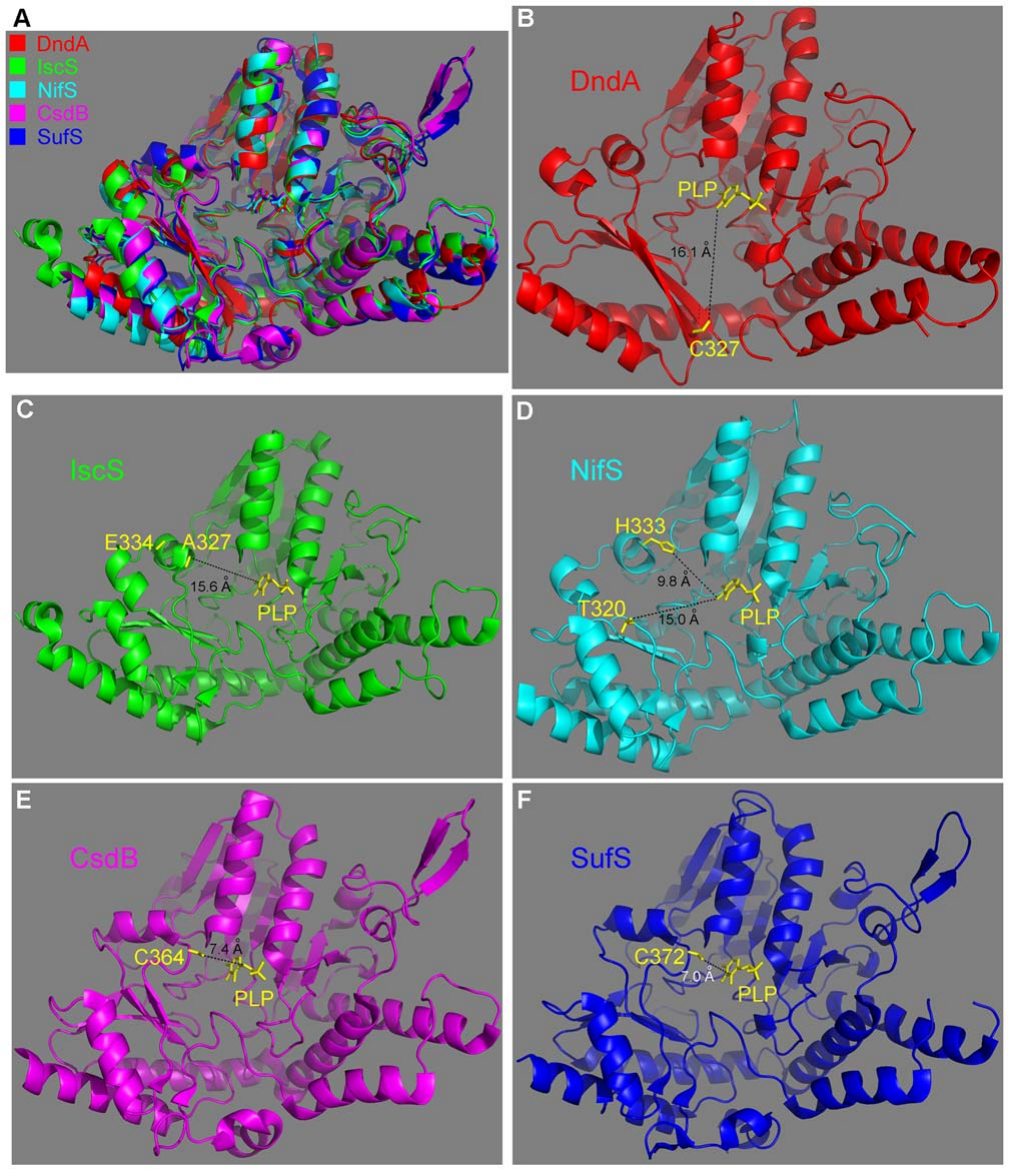
Structural comparison of DndA with related cysteine desulfurases/selenocysteine lyase. (**A**) Structural superimposition of DndA (red), IscS (green, PDB code 1P3W), NifS (cyan, PDB code 1ECX), CsdB (magenta, PDB code 1C0N), and SufS (blue, PDB code 1T3I). Their bound PLP's are shown as sticks. (**B**) In DndA, the active site Cys327 is located on a β strand, and its distance from PLP is ∼16 Å. In IscS (**C**) and NifS (**D**), the active site cysteines are located on relatively long loops, and are not visible in the crystal structure. Visible residues closest to the catalytic cysteines on the primary sequence are no less than 9 Å from PLP. In CsdB (**E**) and SufS (**F**), the active site cysteines are located on relatively short loops, and are ∼7 Å from PLP.

The crystal structure of DndA is similar to those of other cysteine desulfurases/selenocysteine lyase, with root-mean-square deviation (RMSD) of 1.166 Å over 266 Cα atoms with NifS (37.1% sequence identity), 1.267 Å over 276 Cα atoms with IscS (46.6% identity), 1.773 Å over 206 Cα atoms with SufS (24.7% identity), and 1.800 Å over 216 Cα atoms with CsdB (25.6% identity) (Figure 5A). The active site cysteines of IscS (Cys328) and of NifS (Cys324) exist on long and flexible loops. Unfortunately, parts of these loops containing the catalytic cysteines (residues 328–333 of IscS, and residues 321–332 of NifS) are disordered in the crystal structures. The distance between PLP and the residues closest to the catalytic cysteines in their primary sequences (Ala327 and Glu334 in IscS, Thr320 and His333 in NifS) are all no less than 9 Å (Figure 5C and 5D). On the other hand, the active site cysteines of CsdB (Cys364) and of SufS (Cys372) are located on relatively shorter loops, which are visible in the crystal structures. The distances between PLP and these catalytic cysteines are ∼7 Å (Figure 5E and 5F). Although this distance is much shorter than those in IscS and NifS, it is still greater than expected for the nucleophilic attack of the thiolate group of catalytic cysteine on the sulfur atom of the substrate cysteine to occur. In DndA, the distance between the catalytic Cys327 and PLP is about 16 Å, more similar to those in IscS and NifS. The distance between Cys327 and the supposed substrate binding site (the orange circle in Figure 3B) is also greater than 10 Å. Therefore, similar to previously suggested for other cysteine desulfurases [14], a conformational change is required to bring Cys327 to the vicinity of the substrate cysteine for nucleophilic attack. As suggested by our in vitro enzyme activity assay using site-directed mutants, it is unlikely that this conformational change results simply from a random thermal motion.

In order for the catalytic cysteine to carry out the nucleophilic attack on the substrate cysteine, its thiol group needs to be deprotonated first to enhance its nucleophilicity. The residue which performs this task of activating the catalytic cysteine has been proposed to be the histidine stacking on top of the pyridine ring of PLP (His99 in NifS) [15]. This histidine residue is highly conserved, and corresponds to His99 in DndA (Figure 2). Therefore, His99 in DndA might be one candidate that deprotonates the catalytic Cys327. Besides His99, there is another highly conserved basic residue, Arg353, which is closer to Cys327. The distance between Arg353 and Cys327 is 10.5 Å, about half of that (19.9 Å) between His99 and Cys327 (Figure 3B). Thus, Arg353 could be another candidate that activates Cys327.

Up to now, it has not been entirely elucidated what is the difference in molecular basis between cysteine desulfurases and selenocysteine lyases for sulfur/selenium discrimination. Compared to the selenocysteine lyase CsdB, DndA is more similar to cysteine desulfurases IscS and NifS in terms of both sequence identity and structural homology. However, DndA exhibits a higher specific activity for L-selenocysteine over L-cysteine [7]. Elucidation of the mechanistic basis for L-selenocysteine/L-cysteine substrate differentiation would await further investigation.

## Materials and Methods

### Protein Expression and Purification

The cDNA encoding full length wild type (WT) *Streptomyces lividans* DndA (residues 1–380) was cloned into the pET28a (Novagen) vector, with an N-terminal His-tag. Point mutation of C327S on DndA was introduced by the overlapping PCR method. Successfully constructed plasmids were further confirmed by DNA sequencing.

Both WT and C327S mutant DndA proteins were overexpressed in *E. coli* strain BL21(DE3). Cells were grown at 37°C in Luria Broth medium to an OD_600_ of 0.6–0.8, and were then induced overnight at 16°C with 0.2 mM IPTG. Cells were harvested by centrifugation, and resuspended in the binding buffer (25 mM Tris-HCl, pH 8.0, 300 mM NaCl, and 20 mM imidazole). Harvested cells were lysed by sonication, followed by centrifugation. Supernatant of the cell lysate was then purified by Ni^2+^-NTA affinity chromatography (Qiagen), Source 30Q anion exchange chromatography (GE Healthcare), and Superdex 200 gel filtration chromatography (GE Healthcare). The Superdex 200 buffer contained 25 mM Tris-HCl, pH 8.0, 150 mM NaCl, and 2 mM dithiothreitol (DTT). Peak fractions were combined and concentrated to 15 mg/ml, flash-frozen in liquid nitrogen, and stored in −80°C until use.

### Crystallization and structure determination

Crystallization trials for both WT and C327S mutant DndA were performed at 14°C by the hanging-drop vapor-diffusion method, either in the presence or absence of the substrate L-cysteine. Despite considerable effort, we were only able to obtain crystals of C327S mutant DndA in the absence of L-cysteine. The crystallization condition was 20% PEG3,350, 0.1 M sodium acetate, pH 5.5, and 0.2 M ammonium citrate. X-ray diffraction data sets of DndA-C327S were collected at the beamline BL17U1 at Shanghai Synchrotron Radiation Facility (China), using an ADSC Quantum 315r CCD area detector. The diffraction data were processed using the CCP4 program MOSFLM [21].

Crystals of the DndA-C327S protein belonged to the *P*2 space group, with one dimer of DndA-C327S with covalently bound PLP molecules in each asymmetry unit. The structure was determined to 2.4 Å, by the method of molecular replacement with the CCP4 program PHASER [21], using the structure of *E. coli* IscS (PDB code: 1P3W) as the searching model. Model building was performed using COOT [22]. After refinement by the REFMAC program of CCP4 [21], the model has an R factor of 19.2% and R_free_ of 23.4%. The model quality was validated with the CCP4 program PROCHECK [21]. The final model includes residues 2–359 of DndA together with a covalently bound cofactor PLP. In the Ramachandran plot, 97.9% and 2.1% of residues are in the most favored and generally allowed regions, respectively.

### Site-directed mutagenesis

Double point mutants of DndA, S325C/C327S and C327S/S329C, were generated by the overlapping PCR method. Identities of successfully-made constructs were verified by DNA sequencing. Proteins of these two double point mutants were purified as above.

### In vitro cysteine desulfurase activity assay

The cysteine desulfurase activity of DndA was measured as sulfide production using L-cysteine as substrate. The assay was carried out in a mixture with a total volume of 0.8 ml, containing 50 mM Tris pH 8.0, 0.02 mM PLP, 5 mM DTT, and various concentrations of DndA. Reactions were initiated with the addition of 2.5 mM L-cysteine. After 20 minutes incubation at 37°C, the reaction was stopped by adding 100 µl of 20 mM N,N-dimethyl-p-phenylenediamine sulfate in 7.2 M HCl and 100 µl of 30 mM FeCl_3_ in 1.2 M HCl. After further incubation for 30 minutes in the dark, the absorption of methylene blue was measured at 650 nm. The sulfide concentration was determined based on a standard sodium sulfide curve. Specific activities of WT and mutant DndA proteins were expressed as units per milligram of protein, with one unit of enzyme defined as the amount that catalyzed the formation of one nanomole of product in one minute.

### Molecular Graphics

All protein structure figures were generated using PyMOL (http://pymol.sourceforge.net).

### Accession codes

The atomic coordinate and structure factor of the DndA protein with its covalently bound cofactor PLP have been deposited in the Protein Data Bank with the accession number 3VAX.
